# A Rare Case of Functioning Adrenocortical Oncocytoma Presenting as Cushing Syndrome

**DOI:** 10.1155/2016/8964070

**Published:** 2016-02-18

**Authors:** Nicola Tartaglia, Pasquale Cianci, Amedeo Altamura, Vincenzo Lizzi, Fernanda Vovola, Alberto Fersini, Antonio Ambrosi, Vincenzo Neri

**Affiliations:** Department of Medical and Surgical Sciences, University of Foggia, Luigi Pinto Street, No. 1, 71122 Foggia, Italy

## Abstract

Functioning adrenocortical oncocytoma is very rare neoplasm. It is usually nonfunctional and benign and incidentally detected. Generally, these tumors originate in the kidneys, thyroid, parathyroid, and salivary or pituitary glands; they have also been reported in other sites including choroid plexus, respiratory tract, and larynx. Histologically, they are characterized by cells with eosinophilic granular cytoplasm and numerous packed mitochondria. We reported a case of a 44-year-old female who presented with Cushing syndrome for hypersecretion of cortisol due to adrenocortical oncocytoma. Magnetic resonance of abdomen revealed a right adrenal mass. Laparoscopic adrenalectomy was performed and the tumor was pathologically confirmed as benign adrenocortical oncocytoma. After surgical treatment, Cushing's syndrome resolved.

## 1. Introduction

Adrenocortical oncocytoma is a very uncommon neoplasm, usually considered as benign and nonfunctional. Oncocytoma is, in the majority of cases, a benign tumor originating in the kidneys, thyroid, parathyroid, and salivary or pituitary glands [1, 2]; it has also been reported in other sites including choroid plexus [3], respiratory tract [4], and larynx [5]. When secretion is noted, it produces steroid excess with clinical features such as virilization, feminization, and Cushing's syndrome [6]. Histologically, this tumor is characterized by large eosinophilic cells due to the presence of numerous mitochondria [7]. Since the first description of adrenal oncocytoma by Kakimoto in 1986 [8], 49 cases of these secreting tumors until today have been reported in the literature. There are no pathognomonic findings on radiological study, but MR is essential for the detection of enlarged lymph nodes suggestive of malignancy [9]. The diagnosis of these neoplasm is essentially based on histological and immunohistochemical analysis. We describe a case of functioning adrenocortical oncocytoma presenting as Cushing's syndrome.

## 2. Case Presentation

**Figure 1:**
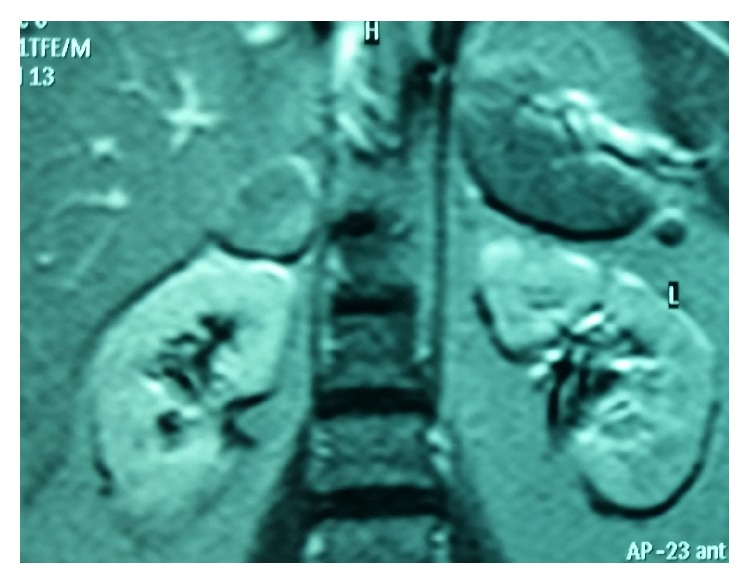
MR of abdomen image (Phase T1 TFE/M) describes an expansive lesion in the lodge of the right adrenal gland that has close relationship with the posterior edge of the adrenal gland.

**Figure 2:**
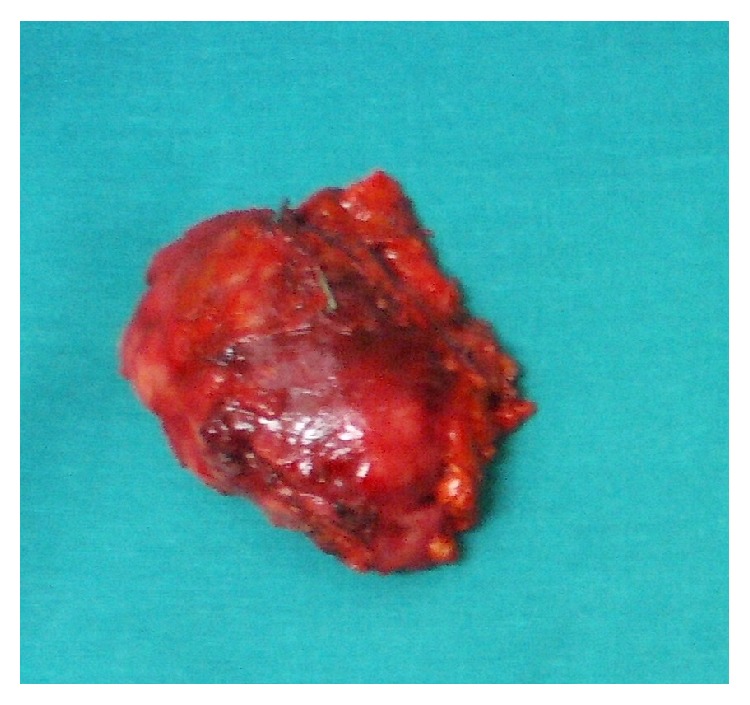
Surgical specimen of the removed right adrenal gland.

**Figure 3:**
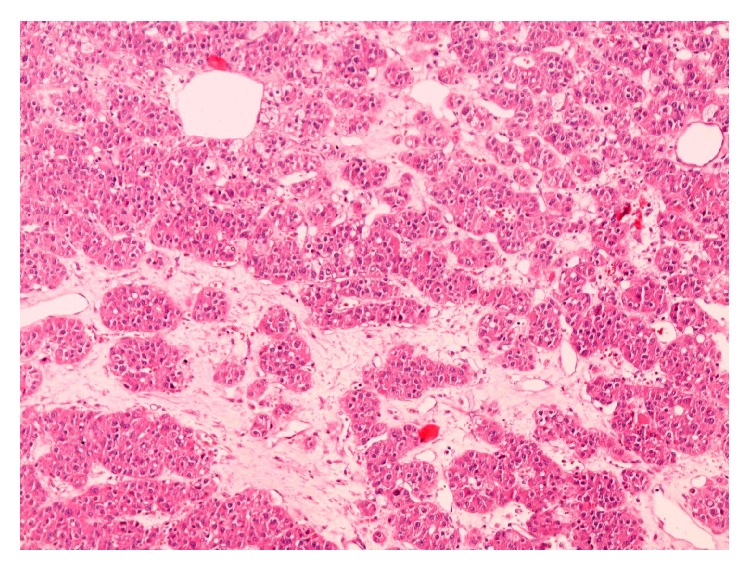
Histopathological examination shows polygonal oncocytic cells arranged in alveolar nests (HE, original magnification 100x).

**Figure 4:**
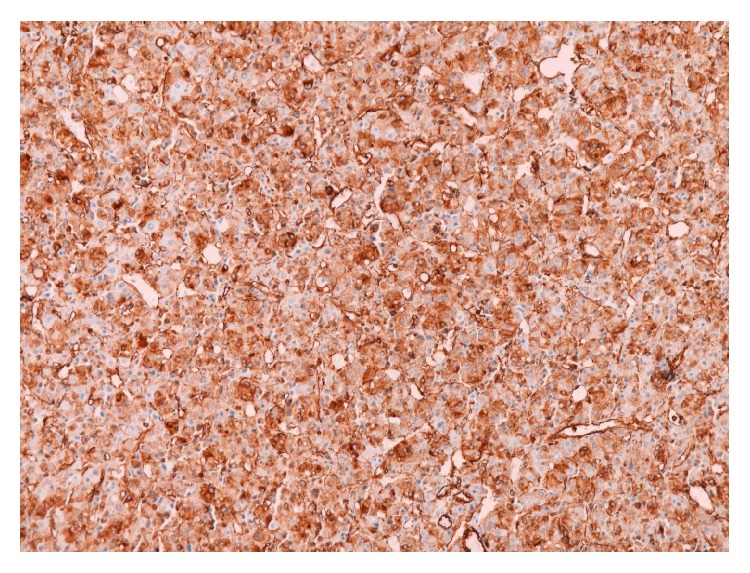
Immunohistochemical examination shows neoplastic cells with diffuse vimentin expression (original magnification 100x).

A 44-year-old woman was admitted to our department presenting with sign and symptoms of Cushing's syndrome since about three years. The patient was a nonsmoker and nonalcoholic. Cardiovascular diseases were high blood pressure and hypertensive cardiopathy treated with *β*-blockers, sartans, thiazide-like diuretic, calcium antagonists, *α*1-blockers, and salicylate drugs. Metabolic disorders were diabetes mellitus type 2 and dyslipidemia treated with metformin, insulin, and statins, respectively. The patient underwent laparoscopic cholecystectomy for lithiasis five years before. Physical examination revealed truncal obesity with moon face and dorsal fat pad (buffalo hump) and thin extremities. The skin showed spontaneous ecchymosis and purple striae on the abdomen. Another sign was hirsutism with increased facial hair. Muscles were hypotonic and hypotrophic and bilateral pretibial edema was present. BMI was 36,8 with a waist circumference of 133 cm. On admission, blood analyses were all within the normal values. The hormonal studies showed a normal value of adrenocorticotropic hormone (ACTH) and circadian rhythm of cortisol. The cortisol level was raised after dexamethasone suppression test (DST) with single dose of this steroid medication (1 mg) and the value was 16,6 *μ*g/dL (normal range < 0,8). Dehydroepiandrosterone-sulfate (DHEA-S), androstenedione (ADS), testosterone, progesterone (P4), and 17-hydroxyprogesterone (17-OHP) were normal. Blood and urine tests for adrenal medulla function and viral and tumor markers were within the normal ranges. MR of the abdomen was performed with intravenous paramagnetic contrast medium. It described an expansive lesion in the lodge of the right adrenal gland, measuring about 3 cm in maximum diameter; this mass had close relations with the posterior edge of the adrenal gland, from which it appeared to originate (Figure 1). The signal was enough homogeneous and after intravenous gadolinium it showed faint enhancement. Based on the clinical manifestations and radiological/hormonal findings, the provisional diagnosis of Cushing's syndrome in a patient with right adrenal adenoma was made. Right laparoscopic adrenalectomy was performed with lateral transperitoneal approach. It was dissected into right triangular ligament and right coronary ligament were dissected in order to mobilize liver and then to identify right middle adrenal vein that was sectioned between clips. Subsequently, the adrenalectomy was carried out. Macroscopic examination revealed the adrenal gland measuring 4 × 4 × 0,5 cm in diameter and weighting 24 gr., including the lesion with nodular form (Figure 2). On cut section, the mass presented a sallow complexion. Microscopic examination showed an oncocytic neoplasm originating in the adrenal cortex. Immunohistochemical analysis demonstrated that tumor cells were diffusely immunopositive for mitochondria (Figure 3), vimentin (Figure 4), and calretinin, while the search for chromogranin was negative. The patient had an uneventful postoperative course and was discharged five days after the operation. During a follow-up period of twelve months after operative excision, the patient was well with no clinical or radiologic evidence of recurrence. The features of Cushing's syndrome receded already in follow-up after six months.

## 3. Discussion

Adrenocortical masses are often detected incidentally by imaging studies during the evaluation for unrelated problem. These “incidentalomas” can be cortical adenomas and carcinomas, cysts, myelolipomas, ganglioneuromas, pheochromocytomas, and adrenal metastases [10]. Adrenal oncocytomas are very rare neoplasm and the term oncocytoma describes a tumor composed predominantly of large cells with abundant and granular eosinophilic cytoplasm for the presence of numerous mitochondria [1, 11]. Generally, these tumors are benign and nonfunctioning, but it is possible to find secreting and malignant tumors which are even more rare. In fact, the exact overall incidence is unknown. We performed a research for secreting oncocytoma using PubMed and Google Scholar and results found were 49 cases from 1991 to 2015. For each of them, there was a hormonal hypersecretion. ACTH, DHEA-S, ADS, testosterone, progesterone, cortisol, aldosterone, estradiol, 17-hydroxyprogesterone (17-OHP), IL-6, epinephrine, and norepinephrine were overproduced individually or in combination. In addition to all other adrenal masses, lesion's size and/or function are the two most important peculiarities of oncocytic neoplasm. Therefore, biochemical analysis and instrumental investigations have to be performed preoperatively. In this time, the major clinical problem is to differentiate benign lesions from malignant ones [12]. Usually, radiologic findings of benign masses are small size, well-defined fibrous capsule without central areas of necrosis, but often enhanced CT scan and MR do not allow a definitive preoperative diagnosis [13]: sometimes, the imaging features of benign and malignant oncocytoma are the same for the lack of pathognomonic features on radiological examinations. For example, the classic central radiating scar that has been described in renal oncocytomas, which are the most widely studied, is not present in adrenal ones. Both in benign and in malignant variants, we could detect, with CT or MR imaging, fibrous encapsulation. Large size more than 6 cm, heterogeneous appearance, and presence of necrosis or calcification in the tumor pose the doubt for a differential diagnosis with the most likely adrenocortical carcinoma. Generally, fat concentration is useful to differentiate the majority of malignant and benign adrenal lesions because almost all malignant lesions are lipid-poor, whereas the majority of benign lesions are lipid-rich and present lower attenuation on CT scan. Recently, it has been noted that injected contrast material tends to wash out of benign lesions faster than malignant ones [14]. Sometimes, a preoperative percutaneous biopsy by CT or US scan guidance provides an accurate diagnosis in some cases of indeterminate mass [15]. Newly, MR imaging has become increasingly useful in characterizing adrenal masses: the chemical shift allows noninvasive distinction between the fat content of adenomas and the lack of fat within metastases. The lack of lipid is not typical of adrenocortical cells but may be secondary to the large amount of mitochondria, which instead fill the cytoplasm of oncocytic cells [16], according to pathologic findings. In 2004, Bisceglia et al. developed the Lin-Weis-Bisceglia criteria for a malignancy score system [17]. The three major criteria are high mitotic rate (>5 mitoses per 50 HPF), atypical mitoses, and venous invasion. The four minor criteria are tumor size >10 cm or weight >200 g, tumor necrosis, capsular invasion, and sinusoidal invasion. The existence of at least one major criterion defines a malignant oncocytoma, the presence of at least one minor criterion defines a borderline oncocytoma, and the absence of all criteria indicates benignancy. There is a variant of adrenal oncocytic neoplasm that grows from adrenal medulla [18]. Morphologic distinction between adrenocortical and medullary tumors can be difficult [19] and the immunohistochemical research becomes important in these cases. Some authors [20] have verified, analyzing cortical and medullary lesions, that 89% were calretinin-positive and 100% were chromogranin-negative from cortical ones. Among pheochromocytomas analyzed, 100% were positive for synaptophysin and chromogranin and all of them were negative for calretinin. So, chromogranin has high sensitivity and specificity like a marker for pheochromocytoma; calretinin, instead, is useful to differentiate tumors of the cortex from those of the medulla. About vimentin immunoreactivity, it was variable, but in most of the cases an oncocytic tumor has diffuse positivity for vimentin. In our case, a 44-year-old woman presented clinical features of Cushing's syndrome. The tumor was about 3 cm in maximum diameter to MR imaging with a homogeneous signal. So, we considered this tumor as benign because none of the criteria were present. After surgical management, histopathological examinations showed that the real size of neoplasm in our patient was 3,5 cm and this confirmed that small oncocytomas are commonly benign tumors. In fact, the lesion was positive for vimentin and calretinin but negative for chromogranin. The immunohistochemical profile was suggestive for adrenocortical oncocytoma and clinical and biological features confirmed its secretory capacity. In conclusion, oncocytic tumor is a histological subtype of all detected adrenal neoplasm. It is uncommon, usually large, benign, and nonfunctional, with prevalence in young women and good prognosis [7, 14]. The surgical treatment for functioning adrenal oncocytoma is often considered inevitable because the imaging findings do not allow its differentiation from adrenocortical carcinoma [13] and in order to eliminate the symptoms due to the hormonal secretion. Generally, the laparoscopic approach can be safely performed in well-experienced surgeons when CT and MR findings reveal a well-encapsulated tumor, but large size, invasion into surrounding tissues; regional adenopathy could be considered exclusion criteria for minimally invasive operation. The diagnosis, clinical behavior, and prognosis of oncocytoma are established by histologic and immunohistochemical investigations. These parameters, in combination with clinical and biochemical ones, can solve also an important practical problem, to discriminate adrenocortical adenoma, carcinoma, or pheochromocytoma.

## References

[1] Chang A., Harawi S. J. (1992). Oncocytes, oncocytosis, and oncocytic tumors. *Pathology Annual*.

[2] Tallini G. (1998). Oncocytic tumours. *Virchows Archiv*.

[3] Sav A., Scheithauer B. W., Mazzola C. A., Ketterling S. R. P., Thompson S. J., Reilly M. H. (2010). Oncocytic choroid plexus carcinoma: case report. *Clinical Neuropathology*.

[4] Tsuta K., Kalhor N., Raso M. G., Wistuba I. I., Moran C. A. (2011). Oncocytic neuroendocrine tumors of the lung: histopathologic spectrum and immunohistochemical analysis of 15 cases. *Human Pathology*.

[5] Kozakiewicz J., Teodorowicz E., Haczyńska-Partyka A., Myrcik G., Lange D., Szwabowicz M. (2007). Adenoma oxyphillicum an extremely rare case of tumour of the larynx end cancer lungs which are going together. *Otolaryngologia Polska*.

[6] Erlandson R. A., Reuter V. E. (1991). Oncocytic adrenal cortical adenoma. *Ultrastructural Pathology*.

[7] Sasano H., Suzuki T., Sano T., Kameya T., Sasano N., Nagura H. (1991). Adrenocortical oncocytoma. A true nonfunctioning adrenocortical tumor. *American Journal of Surgical Pathology*.

[8] Kakimoto S., Yushita Y., Sanefuji T. (1986). Non-hormonal adrenocortical adenoma with oncocytoma-like appearances. *Hinyokika Kiyo*.

[9] Zderic S. A. (2004). Renal and adrenal tumors in children. *Urologic Clinics of North America*.

[10] Sharma D., Sharma S., Jhobta A., Sood R. G. (2012). Virilizing adrenal oncocytoma. *Journal of Clinical Imaging Science*.

[11] Hamperl H. (1950). Oncocytes and the so-called Hurtle cell tumor. *Archives of Pathology*.

[12] Tahar G. T., Nejib K. N., Sadok S. S., Rachid L. M. M. (2008). Adrenocortical oncocytoma: a case report and review of literature. *Journal of Pediatric Surgery*.

[13] Shah R. K., Oto A., Ozkan O. S. (2004). Adrenal oncocytoma: US and CT findings. *JBR-BTR*.

[14] Mearini L., Del Sordo R., Costantini E., Nunzi E., Porena M. (2013). Adrenal oncocytic neoplasm: a systematic review. *Urologia Internationalis*.

[15] Osman Y., El-Mekresh M., Gomha A.-M. (2010). Percutaneous adrenal biopsy for indeterminate adrenal lesion: complications and diagnostic accuracy. *Urologia Internationalis*.

[16] Gandras E. J., Schwartz L. H., Panicek D. M., Levi G. (1996). Case report. Adrenocortical oncocytoma: CT and MRI findings. *Journal of Computer Assisted Tomography*.

[17] Bisceglia M., Ludovico O., Di Mattia A. (2004). Adrenocortical oncocytic tumors: report of 10 cases and review of the literature. *International Journal of Surgical Pathology*.

[18] Chisté M., Poppiti R. J., Bianco F. J. (2013). Oncocytoma of the adrenal gland medulla. *Annals of Diagnostic Pathology*.

[19] Begin L. R. (1992). Adrenocortical oncocytoma: case report with immunocytochemical and ultrastructuraly study. *Virchows Archiv A. Pathological Anatomy and Histopathology*.

[20] Sangoi R. S., McKenney J. K. (2010). A tissue microarray-based comparative analysis of novel and traditional immunohistochemical markers in the distinction between adrenal cortical lesions and pheochromocytoma. *The American Journal of Surgical Pathology*.

